# Online soft measurement method for chemical oxygen demand based on CNN-BiLSTM-Attention algorithm

**DOI:** 10.1371/journal.pone.0305216

**Published:** 2024-06-28

**Authors:** Libo Liu, Xueyong Tian, Yongguang Ma, Wenxia Lu, Yuanqing Luo

**Affiliations:** School of Environmental and Chemical Engineering, Shenyang University of Technology, Shenyang, China; National Institute of Technology Rourkela, INDIA

## Abstract

The measurement of chemical oxygen demand (COD) is very important in the process of sewage treatment. The value of COD reflects the effectiveness and trend of sewage treatment to a certain extent, but obtaining accurate data requires high cost and labor intensity. To1 solve this problem, this paper proposes an online soft measurement method for COD based on Convolutional Neural Network-Bidirectional Long Short-Term Memory Network-Attention Mechanism (CNN-BiLSTM-Attention) algorithm. Firstly, by analyzing the mechanism of the aerobic tank stage in the Anaerobic-Anoxic-Oxic (A^2^O) wastewater treatment process, the selection range of input variables was preliminarily determined, and the collected sample dataset was subjected to correlation analysis. Finally, pH, dissolved oxygen (DO), electrical conductivity (EC), and water temperature (T) were determined as input variables for soft measurement prediction of COD.Then, based on the feature extraction ability of CNN and the advantage that BiLSTM is able to capture the backward and forward dependencies in time series data, combined with the attention mechanism that can assign higher weights to the key data, a CNN-BiLSTM-Attention algorithm model was established to soft measure COD in the effluent from the aerobic zone of the A^2^O wastewater treatment process. At the same time, root mean square error (RMSE), mean absolute error (MAE), mean absolute percentage error (MAPE) and coefficient of determination (R^2^) were utilized Three indicators were used to evaluate the model, and the results showed that the model can accurately predict the value of COD and has a high accuracy. At the same time, compared with models such as CNN-LSTM-Attention, CNN-BiLSTM, CNN-LSTM, LSTM, RNN, BP, SVM, XGBoost, and RF etc., the results showed that the CNN-BiLSTM Attention model performed the best, proving the superiority of the algorithm model.The Wilcoxon signed-rank test indicates significant differences between the CNN-BiLSTM-Attention model and other models.

## 1. Introduction

With the continuous advancement of industrial processes, countries around the world are paying more and more attention to water resource protection issues and investing a large amount of resources to support research on water environment pollution monitoring and control [1, 2]. As is well known, Chemical Oxygen Demand (COD) is an important indicator of the degree of water pollution [3]. The most commonly used method for measuring COD at present is rapid digestion spectrophotometry. Although this method has high detection accuracy, it has problems such as long instrument preparation time and complex operation process, which cannot meet the needs of workers to grasp COD values in real time [4]. Evolving soft measurement technology offers an alternative approach to this problem [5, 6].

Soft measurement technology is a method of using computer technology in industrial processes to estimate, predict, and monitor key process variables in real time [7]. These variables are often not directly measurable or can only be obtained through limited, discrete measurement data. The main goal of soft measurement technology is to estimate unknown variables using known process variable data through models and algorithms, in order to achieve process optimization, quality control, and automation [8]. Using soft measurement technology, the mechanism and correlation analysis of COD are carried out, and indicator parameters that can be easily and accurately measured are selected as input variables. A soft measurement model is constructed to achieve soft measurement prediction of COD values [9, 10].

The application of soft measurement technology in sewage treatment process is very extensive. As the core of soft measurement technology, soft measurement models have been extensively studied by domestic and foreign scholars in this field, and various modeling methods have been developed. Among them, the use of neural networks for soft measurement prediction of key indicator parameters in sewage treatment process has become a research hotspot.Zhang et al. employed a modeling approach that combines a three-layer Back Propagation (BP) neural network with support vector machine to predict the chemical oxygen demand (COD) of lake water quality. The experimental results demonstrate the model’s excellent performance and reliable prediction outcomes [11]. Liu et al. utilized least squares support vector Machine (LS-SVM) to establish a prediction model for effluent chemical oxygen demand (COD) in an anaerobic wastewater treatment system [12]. ZHAO et al. introduced a new soft sensing method. In the proposed method, Sparse Principal Component Analysis (SPCA) is used for dimensionality reduction of the dataset, and the soft sensing model is constructed through an improved Extreme Learning Machine (ELM) algorithm. The results show good performance in predicting BOD5 and COD [13]. Wu et al. constructed a water quality prediction model for the Jinjiang River in China based on artificial neural network (ANN), discrete wavelet transform (DWT), and short-term memory (LSTM) techniques. However, due to limited monitoring conditions, water quality prediction can only be performed for a single location [14]. Nair et al. introduced the development, implementation, and validation of a hybrid soft sensor for estimating total phosphorus (TP) and chemical oxygen demand (COD) in the inflow and outflow of full-scale sewage treatment plants, while also equipped with a GUI for visualization. The results showed that soft sensors have great potential for achieving real-time display of parameters within an acceptable accuracy range [15]. Boshnakov et al. constructed a soft sensing method using BP neural network to monitor COD and BOD in effluent during wastewater treatment. The simulation results show that the soft sensing method based on neural networks can accurately estimate state variables and can be used for real-time monitoring of biochemical wastewater treatment processes [16].

Currently, extensive research is being conducted on the prediction of water quality parameters in sewage treatment engineering using neural networks. While most studies have achieved their predictive goals, several issues still persist: (1) Many studies remain at the algorithmic stage, with some algorithms exhibiting relatively low prediction accuracy. (2) Although the input variables selected by most algorithms have high correlation, the actual measurement is difficult, so it is of little significance for real-time measurement and monitoring. (3) Some soft measurement research solely focuses on predicting historical data without realizing real-time predictions.

In order to solve the above problems, this paper constructs a neural network model based on CNN-BiLSTM-Attention algorithm, and uses the model for the soft measurement of COD in the effluent of aerobic zone of the A^2^O wastewater treatment process, which greatly improves the efficiency of COD determination compared with the traditional laboratory method of COD determination and has great significance in the aspects of real-time monitoring of the water quality situation, cost saving, and so on. The model is of great significance in real-time monitoring of water quality and cost saving.

## 2. Data collection and selection of input variables

### 2.1 Source of water quality sample data

**Fig 1:**
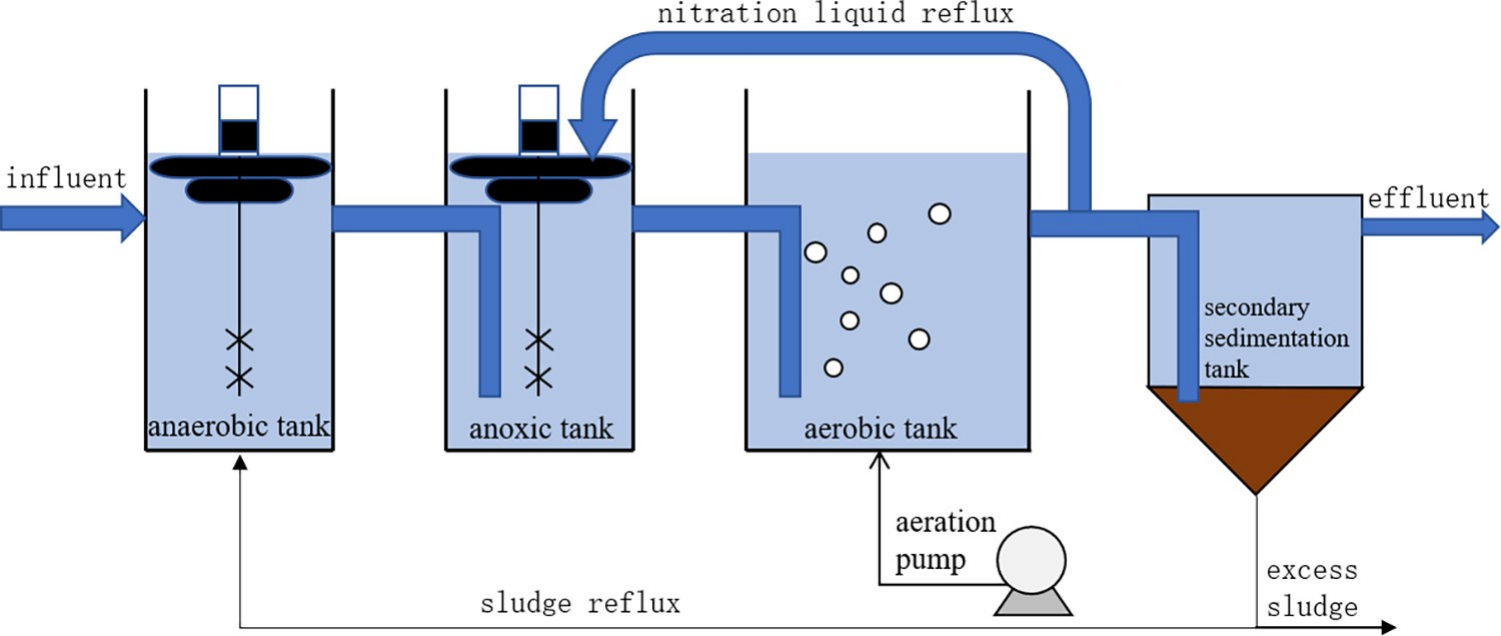
A^2^O method urban sewage treatment simulation device.

The experimental data used in this article comes from the aerobic tank part of an A^2^O method urban sewage treatment simulation device in the laboratory. The schematic diagram of the A^2^O method urban sewage treatment simulation device is shown in Fig 1, which consists of an inlet tank, anaerobic tank, anoxic tank, aerobic tank, secondary sedimentation tank, effluent tank, and internal and external reflux pipelines.

The main processes occurring in the aerobic pool are nitrification of ammonia nitrogen and oxidative decomposition of organic matter:

Ammonia nitrification: When the sewage enters the aerobic pool, nitrifying bacteria in the sludge undergo nitrification under aerobic conditions to convert ammonia nitrogen in the sewage into nitrate, thereby generating a nitrifying liquid. Subsequently, this nitrifying liquid is directed towards the anoxic pool through internal circulation for further reactions.

The oxidative degradation of organic matter: The oxidation and decomposition of organic matter in the water provide energy for phosphorus-absorbing microorganisms, which then absorb phosphorus from the water. Phosphorus is assimilated into the cellular tissue of these microorganisms and subsequently discharged from the system as phosphorus-rich sludge after undergoing precipitation separation.

The aerobic pool of the A^2^O method urban sewage treatment simulation device in this study has a volume of 87.56L. To optimize the reduction of urban domestic sewage, glucose and milk powder were selected as carbon sources, while ammonium sulfate ((NH_4_)_2_SO_4_) was chosen as the nitrogen source for sewage allocation. The sewage was prepared with a carbon-nitrogen ratio of 100:5, ensuring that when COD in the sewage is 500mg/L, the nitrogen content remains at 25mg/L. Table 1 presents the specific materials used for sewage preparation.

**Table 1:** Specific materials for sewage preparation.

	Carbon source		Nitrogen source	
	Raw material(g/L)	COD content(mg/L)	Raw material(g/L)	nitrogen content(mg/L)
**Glucose**	**0.3**	**250**	**-**	
**Milk powder**	**0.3**	**250**	**-**	
**(NH4)2SO4**	**-**	**-**	**1.18**	**25**
**Total**	**-**	**500**	**-**	**25**

### 2.2 Mechanism analysis of COD in aerobic tank effluent

The process of selecting input variables for COD in aerobic tank effluent mainly includes: mechanism analysis of COD in aerobic tank effluent, water quality data collection, and correlation analysis. Firstly, a mechanism analysis is conducted on the COD of the effluent from the aerobic tank of the A^2^O sewage treatment process to preliminarily determine the types of input variables. At the same time, the selection range of input variables should follow the principle of being able to be collected in real-time by sensors, that is, easy to measure. Then, data collection is carried out based on the preliminarily determined input variables. Finally, correlation analysis is used to adjust and screen the input variables, and finally, the input variables are determined.

The key to analyzing the mechanism of COD in the effluent of aerobic tanks in A^2^O sewage treatment process lies in how the process efficiently removes organic pollutants from water. In the anaerobic stage, complex organic matter in wastewater is first decomposed into smaller molecules by anaerobic microorganisms. Next, in the anaerobic stage of denitrification reaction, denitrifying bacteria will use nitrate as an electron acceptor for anaerobic respiration, producing nitrogen gas. At the same time, the oxidation-reduction potential (ORP) of wastewater will decrease, and this reaction process will also be accompanied by a certain amount of organic matter degradation, further reducing the COD content. Finally, in the aerobic stage, which is the main stage for COD removal, nitrifying bacteria use dissolved oxygen (DO) to oxidize and decompose the remaining organic matter, converting it into carbon dioxide and water, thereby significantly reducing the COD concentration in water. At the same time, some inorganic salts such as nitrates are also produced, which can affect the electrical conductivity (EC) of wastewater. The high or low water temperature (T) is not conducive to the growth of microorganisms, which in turn affects the rate of reaction.

According to the above analysis, the indicators that have a significant impact on COD include pH, ORP, EC, DO, and T. Among them, the pH value will affect the microbial activity in the A^2^O process, with an ideal pH of 6–9. Within this range, the efficiency of microbial decomposition of organic matter is higher, thereby reducing COD in water quality. When the pH is too low or too high, it will inhibit the activity of microorganisms and reduce the efficiency of COD removal; The value of ORP has a certain impact on the growth of microorganisms. In anaerobic and anaerobic stages, lower ORP helps promote the growth of certain specific microorganisms, which contribute to the decomposition of organic matter and the removal of nitrogen and phosphorus. In the aerobic stage, higher ORP is conducive to the oxidation and decomposition of organic matter, thereby removing COD; EC is an indicator that reflects the ion concentration in water, indirectly reflecting the content of salt and organic matter in wastewater. Excessive EC may indicate a high concentration of organic matter, which may increase the difficulty of COD removal. Meanwhile, high salinity environments may have inhibitory effects on certain microorganisms, affecting the removal of COD; Water temperature affects the activity of microorganisms and the reaction rate of various reactions, thereby affecting the value of COD; In the aerobic stage, an appropriate level of DO can ensure the rate of nitrification reaction and promote the oxidation and decomposition of organic matter, so dissolved oxygen has a significant impact on COD.

In summary, through the mechanism analysis of COD in aerobic tank effluent, the input variables of COD in aerobic tank effluent were preliminarily determined as pH, ORP, EC, T, and DO.

### 2.3 Collection of water quality sample data

Once the initial selection range of input variables is determined, data collection should be carried out to provide sample data for subsequent correlation analysis and model training. This article uses the A^2^O method urban sewage treatment simulation device mentioned earlier for data collection, and in order to measure the amount of activated sludge in the aeration tank, MLSS collection is added to the data collection process. After 180 days of experimentation, a total of 500 sets of valid data were obtained, and some sample data (collected in March 2023 in Northeast China) are shown in Table 2.

**Table 2:** Partial sample data.

COD(mg/l)	pH	Do(mg/l)	MLSS(g/l)	T(°C)	EC(μs/cm)	ORP(mV)
**38.07**	**7.12**	**7.78**	**3.36**	**18.73**	**1123.68**	**363**
**42.88**	**6.71**	**7.66**	**3.44**	**18.50**	**766.97**	**411**
**65.77**	**7.77**	**8.61**	**2.32**	**18.73**	**942.44**	**312**
**38.11**	**7.14**	**8.05**	**3.78**	**19.13**	**1213.93**	**303**
		**···**				
**38.97**	**7.31**	**7.28**	**3.40**	**19.93**	**1123.68**	**299**
**38.07**	**7.12**	**7.78**	**3.36**	**18.73**	**766.97**	**305**

### 2.4 Selection of input variables

After the data collection is completed, the next step is to analyze these variables by correlation analysis, which is a statistical analysis method to study the correlation between two or more random variables in the same state. Currently, there are three types of correlation coefficients used to represent the correlation between variables: Pearson correlation coefficient, Spearman correlation coefficient and Kendall correlation coefficient. The correlation coefficient can reflect the direction and degree of the trend of change between two variables. The range of values is [–1,1], with 0 indicating no correlation [17]. Positive values indicate positive correlation and negative values indicate negative correlation. The larger the absolute value of the correlation coefficient, the stronger the correlation. By analyzing the process and historical monitoring dataset of the A^2^O wastewater treatment process, the conditions for the applicability of the Pearson correlation coefficient were met and the correlation analysis was performed using SPSS software.

Pearson correlation coefficient is a linear correlation coefficient used to measure the linear relationship between two variables [18, 19]. The Pearson correlation coefficient is calculated as follows:

$$\rho (X,Y)=\frac{\mathrm{cov}(X,Y)}{\sigma X\sigma Y}$$ (1)

Where cov(*X*,*Y*) is the covariance of the variable (*X*,*Y*), *σX* and *σY* are their respective standard deviations.

**Fig 2:**
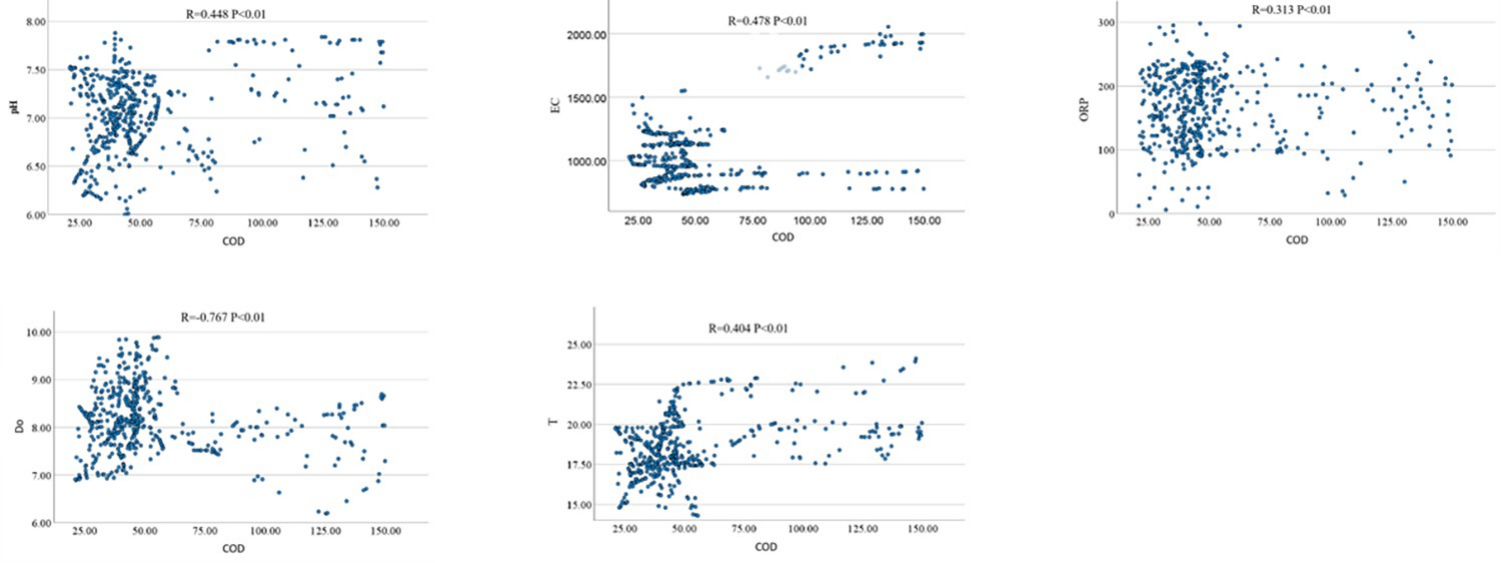
Correlation analysis.

To verify the correlation between COD and the selected input variables, historical monitoring data covering these parameters were selected for correlation analysis. The correlation analysis is shown in Fig 2, and the correlation coefficients are shown in Table 3.

**Table 3:** COD correlation coefficient.

	pH	EC	ORP	DO	T
COD	0.448	0.478	0.313	-0.767	0.404

In order to further verify the rationality of the selected input variables, correlation analysis is conducted between the selected input variables. If the correlation between the selected input variables is strong, it means that there is a lot of duplicate information between the two, and the relevant input variables can be deleted. The correlation coefficients between input variables are shown in Table 4.

**Table 4:** Correlation coefficient between input variables.

	EC	ORP	DO	T
EC	1	0.778	0.417	0.221
ORP	0.778	1	0.112	0.412
DO	0.417	0.112	1	0.317
T	0.221	0.412	0.317	1

According to the analysis results in Table 3, it can be seen that the correlation coefficient between ORP and EC is 0.778, which has a strong correlation, indicating that there is a lot of redundant information between them. At the same time, considering economic and maintenance factors, ORP was removed from the selected input variables, and the final input variables were determined as pH, EC, T, and DO.

### 2.5 Data normalization

The essence of model training is to find a minimum loss function through a large amount of training data. This study involves multiple variables, which have different physical meanings and dimensions. If normalization is not carried out, it will have a significant impact on the training of the model. Therefore, before inputting sample data into the model, all variables in 500 sets of sample data need to be normalized and scaled to a certain ratio between 0 and 1 [20]. By normalizing, the training time is reduced, the convergence speed of the model is accelerated, and the prediction accuracy of the model is improved. The formula for data normalization is as follows:

$$x^{*}=\frac{x-\min x}{\max x-\min x}$$ (2)

Where x* is the result of data normalization, *x* is the sample data, min *x* is the minimum value in the data, and max *x* is the maximum value in the data. Since the input data is normalized, the obtained prediction result is also between 0 and 1, so the final prediction result should be de-normalized when output, and the data de-normalized formula is as follows:

$$x^{*}=y^{*}(x_{\max}-x_{\min})+x_{\min}$$ (3)

Where y* is the predicted value after normalization and x* is the predicted value after de-normalization.

## 3. Modeling method of CNN-BiLSTM-Attention hybrid model

### 3.1 Convolutional neural networks

Convolutional neural network (CNN) is a feedforward neural network with convolutional operation and deep structure. CNN is usually composed of input layer, convolutional layer, pooling layer, fully connected layer, and output layer. Convolutional layers are specialized for data processing, used to filter input data and extract useful information, and use convolutions of different sizes to check the data for convolution [21, 22]. A pooling layer is periodically inserted between consecutive convolution layers to gradually reduce the spatial size of the data body and effectively prevent overfitting. Finally, the training results are obtained through fully connected layers and output layers. A one-dimensional CNN can extract corresponding features of time series. Water quality data belongs to time series; Therefore, one-dimensional CNN networks are applied to feature extraction of wastewater quality data. The process of multidimensional matrix processing for time series data is as follows:

Convolution layer: The preprocessed sewage water quality data is used as input, and the feature matrix is generated by convolution operation.

$$c_{j}^{l}=conv(\sum x_{i}^{l-1}*w_{ij}^{l}+b_{i}^{l})$$ (4)

Where: *conv* is the convolution operation in CNN network; $x_{i}^{l-1}$ and $c_{j}^{1}$ are the input and output of the convolution process, respectively; *l* is the length of the sewage water quality data sequence, *i* and *j* represent the processing position in the convolution process. $w_{ij}^{l}$ is the weight of the convolution layer; $b_{i}^{l}$ is the bias of the convolutional layer.

$$x_{j}^{l}=f(c_{j}^{l})$$ (5)

Where $c_{j}^{l}$ and $x_{j}^{l}$ are the input and output of the activation function respectively; *f* is the nonlinear activation function Re*lu*.

Pooling layer: The pooling operation is performed on the obtained feature matrix.

$$x_{j}^{l+1}=pooling(x_{j}^{1})$$ (6)

The pooling function represents the pooling operation; $x_{j}^{l+1}$ represents the output after the pooling layer.

In this paper, the feature vectors obtained by the CNN network convolution operation are input into the subsequent BiLSTM network layers for time-related feature extraction.

### 3.2 Long Short-Term Memory Neural Networks

**Fig 3:**
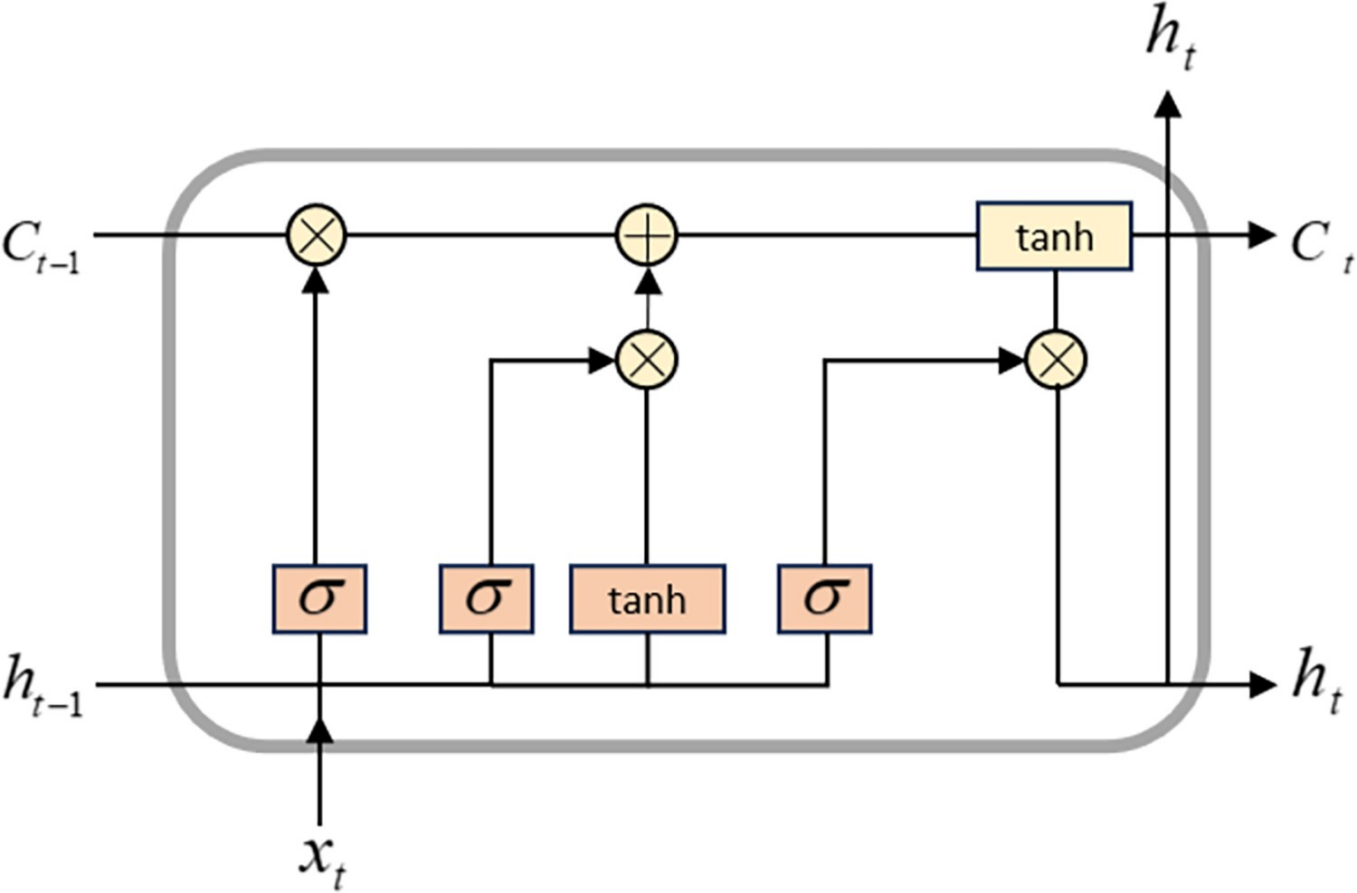
LSTM structure diagram.

Long Short-Term Memory Neural Network (LSTM) is a special type of Recurrent Neural Network (RNN) that has good performance in processing time series data [23, 24]. However, RNN has a long-term dependency problem. When the input time series data is too long, RNN cannot effectively learn long-distance dependency relationships. To solve this problem, researchers combined gating functions and hidden states to propose LSTM [25]. LSTM introduces a "gate" mechanism to control the flow and loss of features, solving the long-term dependency problem of RNN and effectively avoiding problems such as gradient disappearance and explosion [26]. Fig 3 shows the basic structure of LSTM, and the relevant formulas of LSTM are as follows:

1. A forget gate is a hidden cell state in an LSTM that determines the extent to which the previous time’s cell state is retained for the current time.

$$f_{t}=\sigma (W_{f}[h_{t-1},x_{t}]+b_{f})$$ (7)

2. The input gate determines how much of the network’s input at the current time is saved to the unit state.

$$i_{t}=\sigma (W_{i}[h_{t-1},x_{t}]+b_{i})$$ (8)

$${\tilde{C}}_{t}=\tanh(W_{C}[h_{t-1},x_{t}]+b_{C})$$ (9)

3. The update of the storage unit involves discarding unnecessary information and adding new information learned by the network at the current moment.

$$C_{t}=f_{t}\cdot C_{t-1}+i_{t}\cdot {\tilde{C}}_{t}$$ (10)

4. Calculate the output gate and the hidden layer state at the current time

$$o_{t}=\sigma (W_{o}[h_{t-1},x_{t}]+b_{o})$$ (11)

$$h_{t}=o_{t}\cdot \tanh(C_{t})$$ (12)

where, *f*, *i*, *C*, *o* and *h* are the outputs of the forget gate, input gate, cell state, output layer and hidden node, respectively. *σ* and tanh are the *sigmoid* activation function and the hyperbolic tangent function, respectively. *W* and *b* denote the corresponding weight coefficient matrix and bias, respectively.

### 3.3 Bi-directional Long Short-Term Memory Neural Networks

**Fig 4:**
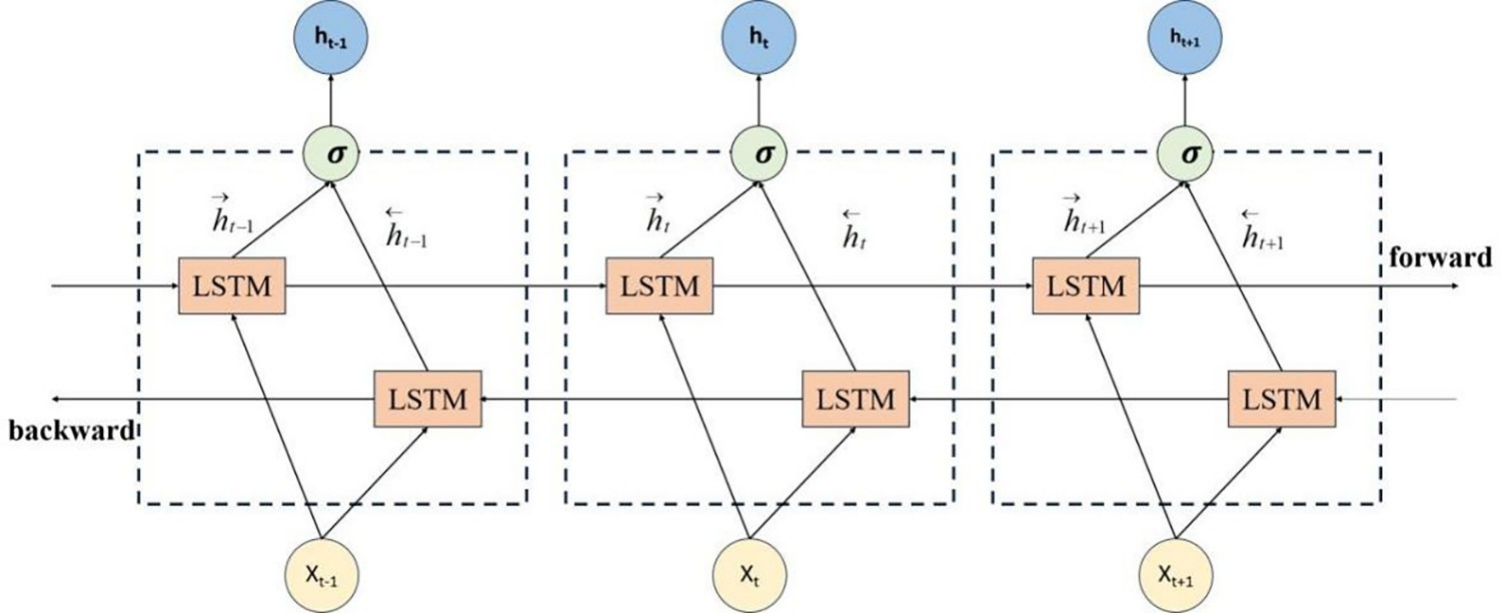
BiLSTM structure diagram.

Bidirectional Long Short-Term Memory Neural Network (BiLSTM) is an optimization and improvement of LSTM, and is a neural network with a powerful architecture. It is formed by combining forward LSTM and backward LSTM, rather than simply increasing the depth of the model through stacking. The hidden state *h*_*t*_ of BiLSTM at the current time *t* includes forward hidden state *h*_*t*−1_ and backward hidden state *h*_*t*+1_. Traditional LSTM can only process data in one direction, so BiLSTM combines reverse LSTM to capture important features that traditional LSTM may miss and fully consider past and future information [27, 28]. Therefore, BiLSTM greatly expands the amount of information available in neural networks, improving the utilization of sample data and prediction accuracy. The structure of BiLSTM is shown in Fig 4.

Where ${\vec{h}}_{t}$ and ${\overset{\leftarrow }{h}}_{t}$ represent the outputs of the forward LSTM and reverse LSTM hidden layers, respectively. The BiLSTM layer generates an output vector Y, where each element is computed as follows.

$$y_{t}=\sigma (\vec{h_{t}},\overset{\leftarrow }{h_{t}})$$ (13)

Where the *σ* function is used to couple sequences $\vec{h_{t}}$ and $\overset{\leftarrow }{h_{t}}$, and the final output of BiLSTM is represented as *Y* = [*y*_1_,*y*_2_,⋯,*y*_*t*_].

### 3.4 Attention mechanism

Attention mechanism originates from the investigation of human vision [29]. For instance, our visual system usually tends to focus on the parts of the image that are helpful for judgment, while ignoring irrelevant information. Different variables have different impacts on the main variable, and important variables contain more critical information, which has a greater impact on the main variable [30]. However, traditional neural networks cannot distinguish the importance of variables, resulting in the inability to highlight important features during model training. Therefore, attention mechanism is introduced for optimization. The attention mechanism assigns different weights based on different features, that is, assigns higher weights to key information, reduces or ignores irrelevant information through weight differentiation, amplifies important information needed, improves processing efficiency and model prediction accuracy. The formula for attention mechanism is as follows:

$$s(x_{i},q)=v^{T}\tanh(Wx_{i}+Uq)$$ (14)

$$\alpha_{i}=soft\max[s(x_{i},q)]$$ (15)

$$att(X,q)=\sum_{i=1}^{N}\alpha_{i}x_{i}$$ (16)

Where *v*, *U* and *W* represent the learnable network parameters, *q* is the query vector, *s*(*x*_*i*_, *q*) is the attention scoring function, and *α*_*i*_ is the attention distribution.

**Fig 5:**
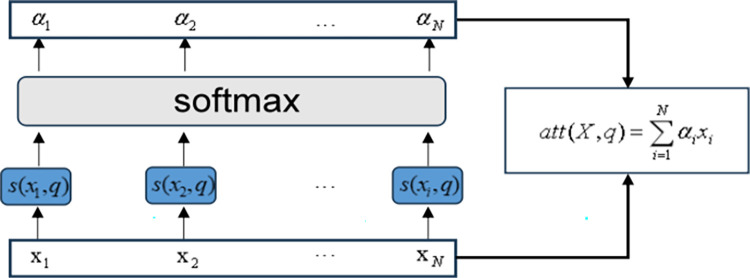
Structural diagram of attention mechanism.

For the input [*x*_1_,*x*_2_⋯*x*_*N*_]of the Attention layer, the correlation between the query vector and each input *x* is calculated through the attention scoring function *s*(*x*_*i*_, *q*) to obtain a score, and then these scores are normalized using the softmax function to obtain a series of attention distributions [*α*_1_,*α*_2_⋯*α*_*N*_]. Finally, the input information is weighted and summed according to the attention distribution to obtain the output sequence *att*(*X*,*q*). The structure of the attention mechanism is shown in Fig 5.

### 3.5 CNN-BiLSTM-Attention algorithm model

**Fig 6:**
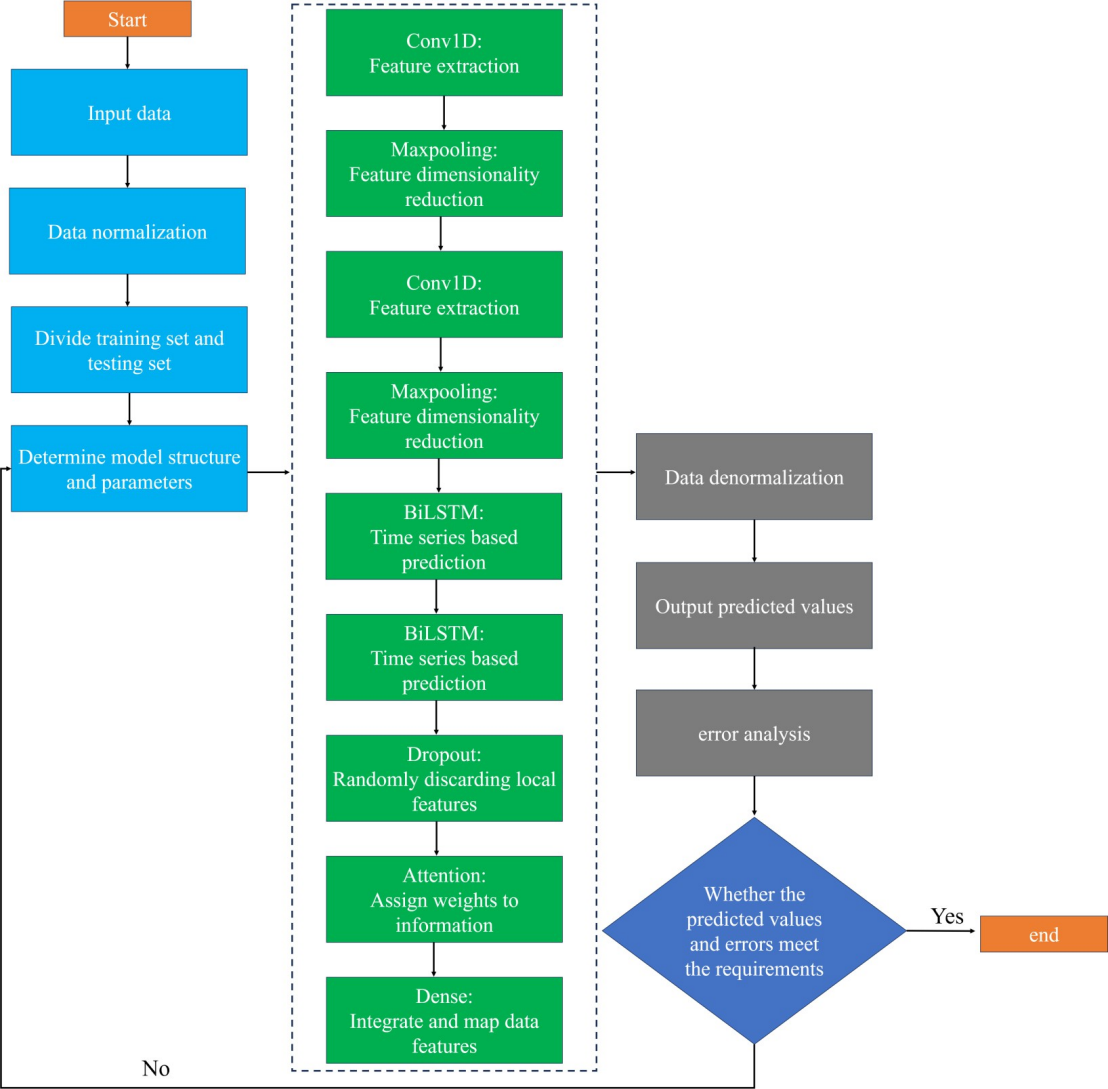
CNN-BiLSTM-Attention model flow chart.

In this paper, a CNN-BiLSTM-Attention algorithm model is proposed and applied to soft-measurement prediction of COD values of effluent from aerobic zone of A^2^O wastewater treatment process. First, the input data are preprocessed, next, Conv1D is used to perform convolution operation on the data to extract the local features of the data. Then, BiLSTM serializes the extracted local features. Meanwhile, during the training process, Dropout layer is utilized to randomly reject some features to improve the robustness of the model. On this basis, the attention mechanism is introduced to weight the sequence data, and different weights are assigned according to the importance of the sequence data. Finally, the predicted values in the test set are output, and the error analysis is given and the model is saved. The flow chart of the model is shown in Fig 6.

### 3.6 Model performance metrics

In order to test the performance of the CNN-BiLSTM-Attention hybrid model, three indexes, root mean square error (RMSE), mean absolute error (MAE), mean absolute percentage error (MAPE), and coefficient of determination (R^2^), were used to evaluate the CNN-BiLSTM-Attention hybrid model. RMSE reflects the degree of deviation between the true value and the predicted value, and MAE represents the average absolute error between the true value and the predicted value. The smaller the RMSE and MAE are, the closer the predicted value is to the true value and the better the prediction effect is, while R^2^ represents the degree of fit, and the closer it is to 1, the better the fitting ability of the data is. The formula for RMSE, MAE, MAPE, and R^2^ is as follows:

$$RMSE=\sqrt{\frac{1}{m}\sum_{i=1}^{m}{\left(y_{i}-{\hat{y}}_{i}\right)}^{2}}$$ (17)

$$MAE=\frac{1}{m}\sum_{i=1}^{m}\left|{\hat{y}}_{i}-y_{i}\right|$$ (18)

$$R^{2}=1-\frac{\sum_{i=1}^{m}{\left({\hat{y}}_{i}-y_{i}\right)}^{2}}{\sum_{i=1}^{m}{\left({\bar{y}}_{i}-y_{i}\right)}^{2}}$$ (19)

$$MAPE=\frac{1}{n}\sum_{i=1}^{n}\left|\frac{y_{i}-y_{i}^{\prime }}{y_{i}}\right|$$ (20)

Where *m* is the number of samples in the water quality sample data, *y*_*i*_ is the actual COD value, ${\hat{y}}_{i}$ is the predicted COD value, *RMSE* is the mean square error, *MAE* is the mean absolute error, *R*^2^ is the coefficient of determination, and MAPE is the average absolute percentage error.

## 4.CNN-BiLSTM-Attention model architecture and performance analysis

### 4.1 Experimental environment

In this paper, the deep learning framework Keras is applied to build the environment required for simulation experiments. The specific environment parameters are: CPU, AMD Ryzen 7 4800H; GPU, NVIDIA GeForce GTX 1650; RAM, 16GB; Operating system, windows 11, 64-bit;keras version 2.8.0; tensorflow version 2.8.0; python version 3.9.

### 4.2 Model parameter setting

In this study, GridSearchCV from scikit-learn library is used to find the best combination of hyperparameters, which provides a convenient interface to define hyperparameter grids, instantiate models, perform cross-validation and performance evaluation. Considering the grid search method in this paper, we define several key hyperparameters, including learning rate, batch size, dropout rate, CNN layer number and convolution kernel parameter, BiLSTM layer number and cell number. At the same time, a reasonable search space is set for each hyperparameter to cover the best possible configuration, as shown in Table 5.

**Table 5:** The hyperparameter search space settings.

Hyperparameter	Search scope
number of CNN layers	[1,2,3]
Filters	[32,64,128]
Kernel Size	[1,3,5]
Number of BiLSTM layers	[1,2,3]
Number of BiLSTM units	[64,128,256,512]
Dropout	[0.1,0.2,0.3,0.4,0.5]
Learning Rate	[0.01.0.001]
Batch Size	[32,64]

During grid search, cross validation is used to evaluate the performance of the model. Cross validation divides the dataset into multiple subsets, and then uses each subset as the test set and the remaining subsets as the training set for model training and validation. This process will be repeated multiple times, each time using a different subset as the test set. Finally, calculate the average of all validation results as the performance indicator for this hyperparameter combination. Through multiple experimental analyses, the optimal values of these parameters were obtained, as shown in Table 6.

**Table 6:** Description of optimal model parameters.

Parameter type	Parameter description
Train set: Test set	8:2
Convolution1D layers	2
Convolution1D parameters	Filters = 128; strides = 4; activation = ’relu’
Max-pooling1D layers	2
Max-pooling1D parameters	Pool size = 1; strides = 4
BiLSTM layers	2
BiLSTM parameters	Units = 512; activation = ’tanh’
Dropout coefficient	0.1
Dense	Units = 1; activation = ’linear’
Learning rate	0.01
Batch_size	64
Epochs	100

### 4.3 Model performance and analysis

**Fig 7:**
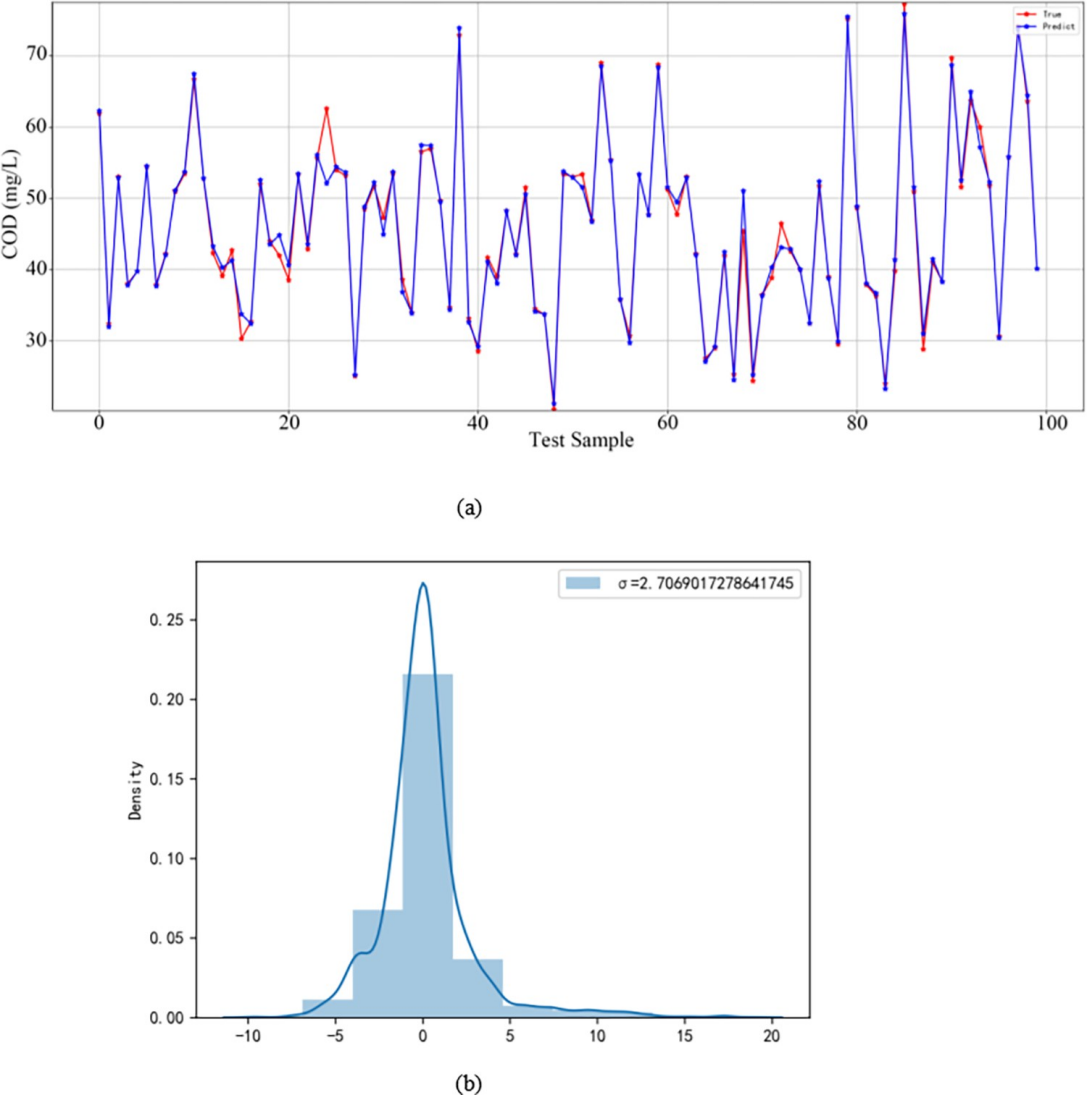
(a) Comparison between true and predicted values in the test set (b) Prediction error distribution density distribution map.

In order to verify the performance of the CNN-BiLSTM-Attention algorithm model, this paper divides 20% of the 500 sample data after data normalization into the test set and 80% into the training set, and trains and tests the model, the model training results are shown in Fig 7. Meanwhile, evaluate the performance of the model using RMSE, MAE, MAPE, and R^2^.

It can be seen from Fig 7 that the model can capture the inherent law of the data well, with high prediction accuracy, small error between the predicted value and the true value, and fast convergence speed.

### 4.4 Comparative validation of different models

**Fig 8:**
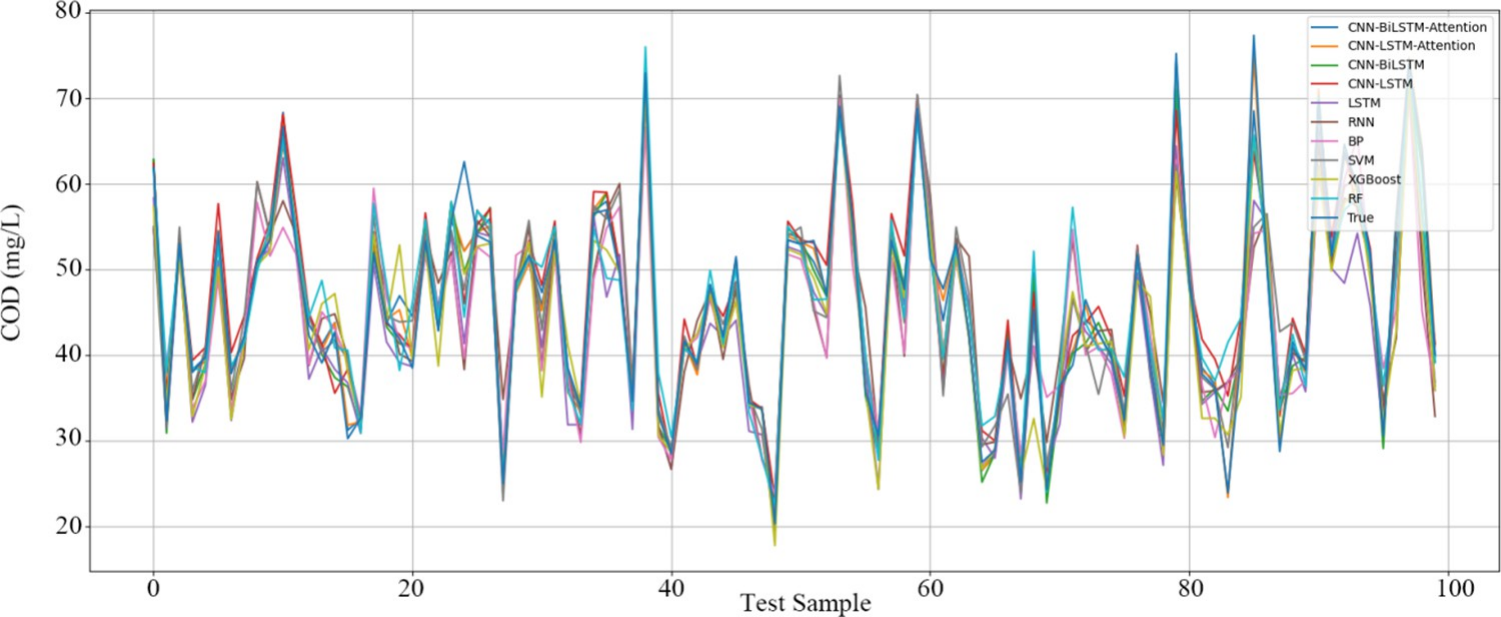
Comparison of COD prediction results.

In order to further verify the performance of CNN-BiLSTM-Attention model, CNN-BiLSTM-Attention model was compared with other models (CNN-LSTM-Attention, CNN-BiLSTM, CNN-LSTM, LSTM, RNN, BP).The comparison of COD prediction results is shown in Fig 8, and RMSE, MAE, MAPE, and R^2^ of each model structure are shown in Table 7.

**Table 7:** Comparison of evaluation indicators for different models.

Model	RMSE	MAE	MAPE	R^2^
**CNN-BiLSTM-Attention**	**0.4001**	**0.2719**	**1.6111**	**0.9796**
**CNN-LSTM-Attention**	**0.4831**	**0.3283**	**1.6982**	**0.9683**
**CNN-BiLSTM**	**0.5209**	**0.3309**	**1.7203**	**0.9611**
**CNN-LSTM**	**0.6709**	**0.5002**	**1.7833**	**0.9454**
**LSTM**	**0.8593**	**0.5403**	**2.0354**	**0.9133**
**RNN**	**1.0884**	**0.6293**	**2.2346**	**0.8604**
**BP**	**1.1315**	**0.7320**	**2.2783**	**0.8435**
**SVM**	**0.9342**	**0.6001**	**2.1240**	**0.8943**
**XGBoost**	**0.9105**	**0.5994**	**2.1151**	**0.8903**
**RF**	**1.1293**	**0.6990**	**2.2620**	**0.8552**

From Fig 8 and Table 7, it can be concluded that the prediction accuracy of the CNN-BiLSTM-Attention model is higher than that of other models. In terms of RMSE, the CNN-BiLSTM-Attention model decreased by 17.18%, 23.19%, 40.36%, 53.44%, 63.24%, 64.64%, 57.17%, 56.06%, and 64.57% compared to the other nine models, respectively. In terms of MAE, the CNN-BiLSTM-Attention model reduced by 18.61%, 22.63%, 45.95%, 51.07%, 58.38%, and 62.16% compared to the other nine models, respectively. In terms of MAPE, the CNN-BiLSTM-Attention model decreased by 5.13%, 6.35%, 9.66%, 20.85%, 27.90%, 29.28%, 24.15%, 23.83%, and 28.78% compared to the other nine models, respectively. In R ^2^ In terms of performance, the CNN-BiLSTM-Attention model has improved by 1.17%, 1.92%, 3.62%, 7.26%, 13.85%, 16.14%, 9.54%, 10.03%, and 12.70% compared to the other nine models, respectively. Through model comparison, the accuracy of the proposed CNN-BiLSTM-Attention model for COD prediction has been further verified, and its performance is superior to other models.

### 4.5 Wilcoxon signed-rank test

In order to consolidate the conclusions of the comparative verification of different models in this chapter, we conducted a non parametric statistical test, namely Wilcoxon signed rank test, on the proposed CNN-BiLSTM-Attention model and CNN-LSTM-Attention model, and CNN-BiLSTM model and CNN-LSTM model. the null hypothesis is these four models are similar, that is, combining convolutional neural networks, bidirectional long short-term memory, and attention mechanisms did not improve the accuracy of soft measurement. Therefore, the soft measurement results of the four models mentioned above are very similar or identical. In Table 8, the average, standard deviation, maximum, and minimum values of various performance evaluation indicators for the four models are presented. The CNN-BiLSTM-Attention model was compared pairwise with other models. According to the Wilcoxon signed rank test, it can be concluded from this table that after 10 independent runs, RMSE of CNN-BiLSTM-Attention model The mean values of MAE, MAPE, and R^2^ are all superior to other models. In Table 9, the positive and negative ranks of each pair of error measures corresponding to the CNN-BiLSTM-Attention model and other models are presented. Further evaluate and analyze the tests in Table 10 based on the descriptive statistical data presented in Tables 8 and 9.

**Table 8:** Descriptive statistics.

Model	N	Mean	Std. Deviation	Minimum	Maximum
**CNN-BiLSTM-Attention_RMSE**	**10**	**0.4334**	**0.0121**	**0.4001**	**0.4921**
**CNN-BiLSTM-Attention_MAE**	**10**	**0.2783**	**0.0036**	**0.2719**	**0.2939**
**CNN-BiLSTM-Attention_MAPE**	**10**	**1.6337**	**0.0102**	**1.6111**	**1.6785**
**CNN-BiLSTM-Attention_R** ^**2**^	**10**	**0.9693**	**0.0031**	**0.9622**	**0.9796**
**CNN-LSTM-Attention_RMSE**	**10**	**0.5002**	**0.0132**	**0.4831**	**0.5199**
**CNN-LSTM-Attention_MAE**	**10**	**0.3689**	**0.0076**	**0.3283**	**0.4138**
**CNN-LSTM-Attention_MAPE**	**10**	**1.7198**	**0.0178**	**1.6982**	**1.7432**
**CNN-LSTM-Attention_R** ^**2**^	**10**	**0.9601**	**0.0042**	**0.9473**	**0.9683**
**CNN-BiLSTM_RMSE**	**10**	**0.5631**	**0.0195**	**0.5209**	**0.6333**
**CNN-BiLSTM_MAE**	**10**	**0.3381**	**0.0093**	**0.3309**	**0.3673**
**CNN-BiLSTM_MAPE**	**10**	**1.7160**	**0.0193**	**1.7203**	**1.8433**
**CNN-BiLSTM_R** ^**2**^	**10**	**0.9522**	**0.0123**	**0.9398**	**0.9611**
**CNN-LSTM_RMSE**	**10**	**0.6760**	**0.0167**	**0.6709**	**0.6811**
**CNN-LSTM_MAE**	**10**	**0.5833**	**0.0118**	**0.5002**	**0.6150**
**CNN-LSTM_MAPE**	**10**	**1.8738**	**0.0235**	**1.7833**	**1.9853**
**CNN-LSTM_R** ^**2**^	**10**	**0.9348**	**0.0158**	**0.9203**	**0.9454**

**Table 9:** Wilcoxon signed-ranks test.

Model Versus	Rank Summary	N
**CNN-BiLSTM-Attention_RMSE—CNN-LSTM-Attention_RMSE**	**Negative Ranks**	**7**
	**Positive Ranks**	**3**
	**Ties**	**0**
	**Total**	**10**
**CNN-BiLSTM-Attention_MAE—CNN-LSTM-Attention_MAE**	**Negative Ranks**	**10**
	**Positive Ranks**	**0**
	**Ties**	**0**
	**Total**	**10**
**CNN-BiLSTM-Attention_MAPE—CNN-LSTM-Attention_MAPE**	**Negative Ranks**	**10**
	**Positive Ranks**	**0**
	**Ties**	**0**
	**Total**	**0**
**CNN-BiLSTM-Attention_R** ^**2**^ **—CNN-LSTM-Attention_R** ^**2**^	**Negative Ranks**	**2**
	**Positive Ranks**	**8**
	**Ties**	**0**
	**Total**	**10**
**CNN-BiLSTM-Attention_RMSE—CNN-BiLSTM_RMSE**	**Negative Ranks**	**10**
	**Positive Ranks**	**0**
	**Ties**	**0**
	**Total**	**10**
**CNN-BiLSTM-Attention_MAE—CNN-BiLSTM_MAE**	**Negative Ranks**	**10**
	**Positive Ranks**	**0**
	**Ties**	**0**
	**Total**	**10**
**CNN-BiLSTM-Attention_MAPE—CNN-BiLSTM_MAPE**	**Negative Ranks**	**10**
	**Positive Ranks**	**0**
	**Ties**	**0**
	**Total**	**10**
**CNN-BiLSTM-Attention_R** ^**2**^ **—CNN-BiLSTM_R** ^**2**^	**Negative Ranks**	**0**
	**Positive Ranks**	**10**
	**Ties**	**0**
	**Total**	**10**
**CNN-BiLSTM-Attention_RMSE—CNN-LSTM_RMSE**	**Negative Ranks**	**10**
	**Positive Ranks**	**0**
	**Ties**	**0**
	**Total**	**10**
**CNN-BiLSTM-Attention_MAE—CNN-LSTM_MAE**	**Negative Ranks**	**10**
	**Positive Ranks**	**0**
	**Ties**	**0**
	**Total**	**10**
**CNN-BiLSTM-Attention_MAPE—CNN-LSTM_MAPE**	**Negative Ranks**	**10**
	**Positive Ranks**	**0**
	**Ties**	**0**
	**Total**	**10**
**CNN-BiLSTM-Attention_R** ^**2**^ **—CNN-LSTM_R** ^**2**^	**Negative Ranks**	**0**
	**Positive Ranks**	**10**
	**Ties**	**0**
	**Total**	**10**

**Table 10:** Test statistics.

Model Versus	Z	Asymp.Sig.(2-tailed)
**CNN-BiLSTM-Attention_RMSE—CNN-LSTM-Attention_RMSE**	**-2.312**	**0.020**
**CNN-BiLSTM-Attention_MAE—CNN-LSTM-Attention_MAE**	**-2.452**	**0.014**
**CNN-BiLSTM-Attention_MAPE—CNN-LSTM-Attention_MAPE**	**-2.617**	**0.008**
**CNN-BiLSTM-Attention_R** ^**2**^ **—CNN-LSTM-Attention_R** ^**2**^	**-2.121**	**0.033**
**CNN-BiLSTM-Attention_RMSE—CNN-BiLSTM_RMSE**	**-3.332**	**0.000**
**CNN-BiLSTM-Attention_MAE—CNN-BiLSTM_MAE**	**-3.348**	**0.000**
**CNN-BiLSTM-Attention_MAPE—CNN-BiLSTM_MAPE**	**-3.421**	**0.000**
**CNN-BiLSTM-Attention_R** ^**2**^ **—CNN-BiLSTM_R** ^**2**^	**-3.444**	**0.000**
**CNN-BiLSTM-Attention_RMSE—CNN-LSTM_RMSE**	**-3.501**	**0.000**
**CNN-BiLSTM-Attention_MAE—CNN-LSTM_MAE**	**-3.683**	**0.000**
**CNN-BiLSTM-Attention_MAPE—CNN-LSTM_MAPE**	**-3.603**	**0.000**
**CNN-BiLSTM-Attention_R** ^**2**^ **—CNN-LSTM_R** ^**2**^	**-3.555**	**0.000**

The Wilcoxon signed rank test shows significant differences among the four models mentioned above, with Z-values of the test statistic being -2.312, -2.452, -2.617, -2.121, -3.332, -3.134, -3.421, -3.109, -3.501, -3.683, -3.603, and -3.555, respectively. From Asymp.Sig.(2-tailed) can infer the relationship between the CNN-BiLSTM-Attention model and the CNN-LSTM-Attention model, RMSE is 0.020, MAE is 0.014, MAPE is 0.008, R^2^ is 0.033. The CNN-BiLSTM-Attention Model and CNN-BiLSTM All indicators between CNN-LSTM are 0. Based on the results of Wilcoxon’s signed-rank test, we reject the null hypothesis, the CNN-BiLSTM-Attention model differs significantly from other models.

## 5. Conclusion

COD is very critical in the wastewater treatment process, but due to the complexity of water quality conditions, accurate prediction is very challenging. Therefore, this paper proposed a soft measurement method of COD based on CNN-BiLSTM-Attention algorithm to predict the COD value in the wastewater treatment process, and verified the accuracy of the model through experiments. It provides a low-cost and efficient method for real-time monitoring of COD in wastewater treatment.

The use of this model combined with auxiliary variable sensors as a measurement method for COD has advantages such as high monitoring efficiency, low labor intensity, and small sampling error compared to traditional laboratory measurement methods. Compared with COD sensors, there are also many advantages, including low cost, short maintenance cycle, and strong environmental adaptability. Especially when the monitoring conditions are poor and the monitoring points are scattered, the advantages of this model can be better reflected.

Although there has been research on this soft measurement method, it still has certain limitations and can be further improved in future research, including: (1) when used in new application scenarios, the model needs to be retrained with historical data from new application scenarios. (2) In the short term, the accuracy of soft measurement can be guaranteed, but over time, if there are significant changes in water quality conditions, the error will increase.

In the future research, the historical data should be saved in real time, and the latest historical data should be used to train the model every once in a while, so as to ensure a high accuracy for a long time and extend the effective prediction period of the model. In addition, new application scenarios and more sample data will be introduced in the future to further verify the performance of the model.

## Supporting information

S1 Table 500 sets of sample data. (XLSX)
